# Comments on “SpatialDG: a novel spatial domain identification method for spatially resolved transcriptomics data based on dual-graph neural network”

**DOI:** 10.1093/bib/bbag607

**Published:** 2026-11-02

**Authors:** Mısra Taşçı, Mehmet Gönen

**Affiliations:** Graduate School of Sciences and Engineering, Koç University, İstanbul 34450, Türkiye; Department of Industrial Engineering, Koç University, College of Engineering, İstanbul 34450, Türkiye; School of Medicine, Koç University, İstanbul 34450, Türkiye

**Keywords:** clustering, machine learning, gene expression

Dear Editor,

In a recent article in *Briefings in Bioinformatics*, a new spatial domain identification method, namely, SpatialDG, has been proposed [1]. The clustering performance of the model has been reported to be significantly better than existing methods in the literature. However, upon closer inspection of the implementation, we noticed that the clustering performance metrics reported were not accurate, but inflated due to label leakage. We have discussed this concern in the following sections with relevant details.

## Spatial domain identification by SpatialDG

Spatial transcriptomics (ST) technologies have advanced rapidly in recent years, enabling researchers to gain a better understanding of tissue biology by providing both gene expression and location information of measured spots on the tissue slice. One principal goal in this field is spatial domain identification, clustering ST spots into continuous areas on biological tissue with similar gene expression patterns. Over 50 methods have been developed for this purpose [2].

SpatialDG [1] is a recently published method that utilizes a dual-graph self-supervised contrastive learning framework to delineate spatial domains in ST data. The method was evaluated on four ST datasets, but the Adjusted Rand Index (ARI) scores were reported for only two of them. The first dataset is the widely used benchmark dorsolateral prefrontal cortex (DLPFC) dataset [3] acquired with the 10x Visium platform. DLPFC dataset includes 12 slices of postmortem human DLPFC tissue from three donors, labeled by white matter and cortical layers 1 to 6. The second dataset contains one slice of human breast cancer tissue, also acquired with 10x Visium [4]. This dataset was annotated by [5] into 20 regions representing areas with different levels of malignancy.

## Clustering performance is inflated due to label leakage

When we examined the code of SpatialDG (https://github.com/Izzilab/SpatialDG), we found that while calculating the ARI scores for the two datasets, the model chooses the highest ARI among different epochs. This is clearly seen between lines 131 and 136 in dlpfctest.py and between lines 129 and 134 in HBC_test.py (accessed 11 September 2026). We reproduced the results and calculated the ARI scores for every epoch for the DLPFC dataset (Fig. 1). For each slice, the maximum ARI achieved corresponded to a different epoch. We compared the reported ARI scores with those obtained at the final epoch, which SpatialDG sets to 80 (Table 1). The mean difference between reported and last-epoch ARI scores among 12 slices was 0.15. The median final-epoch ARI across slices was 0.41, and the reported median ARI was 0.60. For the breast cancer dataset, the reported ARI score was 0.66. When we reproduced the results using the authors' chosen setting of 200 epochs for this dataset, the maximum ARI score across epochs was 0.66, obtained on epoch number 126. The final-epoch ARI was 0.58.

**Table 1:** Comparison of the reported ARI scores with the ARI obtained at the final training epoch for the 12 DLPFC slices.

Slice	Reported ARI	Final-epoch ARI	*Δ * ARI
151507	0.74	0.69	0.05
151508	0.55	0.46	0.09
151509	0.64	0.65	−0.01
151510	0.56	0.54	0.02
151669	0.48	0.30	0.18
151670	0.48	0.34	0.14
151671	0.78	0.55	0.23
151672	0.77	0.57	0.20
151673	0.58	0.37	0.21
151674	0.59	0.37	0.22
151675	0.58	0.32	0.26
151676	0.57	0.34	0.23

**Figure 1:**
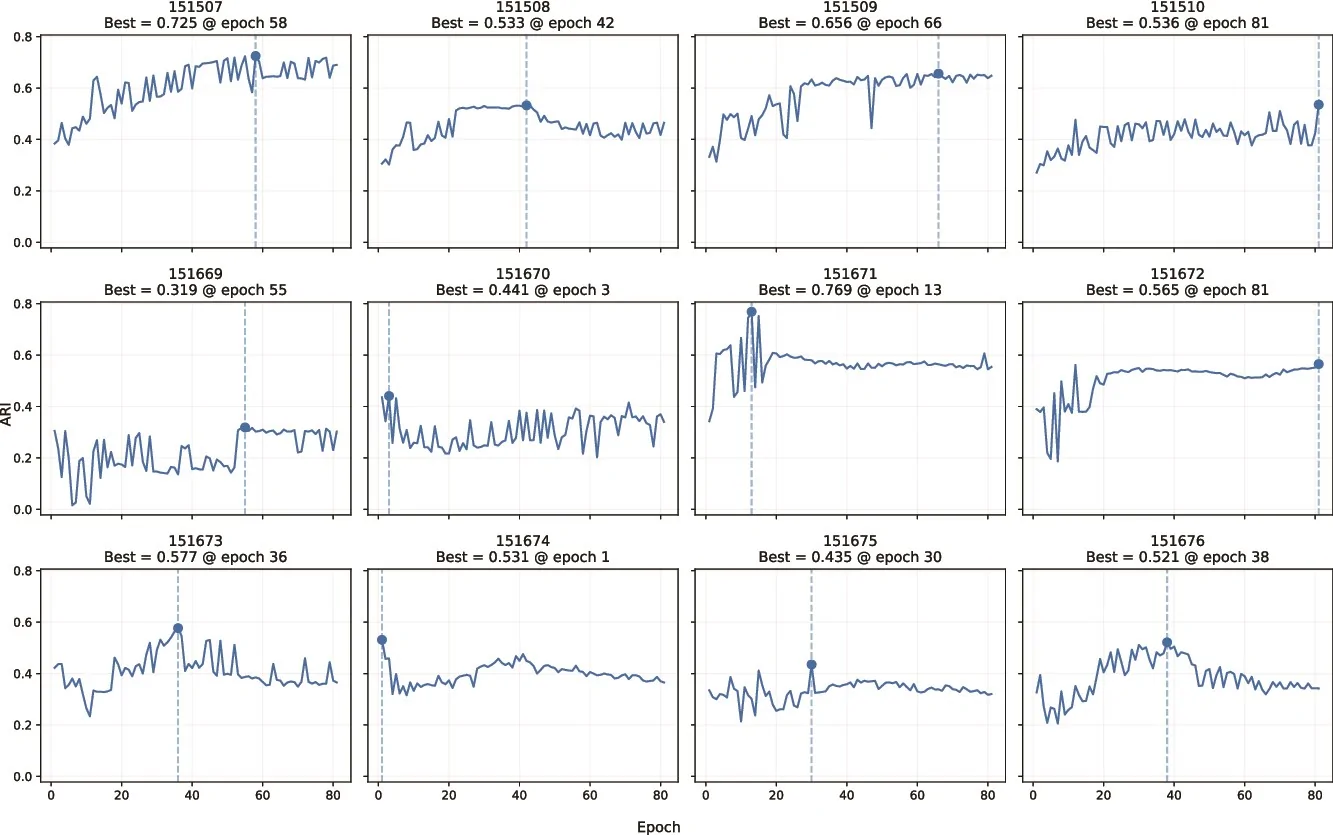
Variability of ARI scores across epochs in SpatialDG for the 12 DLPFC slices. The maximum ARI score and the corresponding epoch are marked for each slice.

The authors also claim that SpatialDG maintains a more consistent performance across slices in the DLPFC dataset than other methods. Since the reported ARI scores are chosen among different epochs based on the ground truth, a lower variance is expected because lower-performing epochs are replaced with the best-performing ones. The article reports ARIs of three representative slices 151671, 151673, and 151676 with five different seeds. We calculated the final-epoch ARI scores for these slices across the same seeds (Table 2). The mean final-epoch ARI across five seeds was lower than the reported mean and standard deviations were higher.

**Table 2:** Comparison of reported ARI (selected based on ground truth) and final-epoch ARI across five random seeds for three DLPFC slices. The values in the final rows are mean and standard deviation across seeds.

Seed	151671		151673		151676	
	Reported	Final	Reported	Final	Reported	Final
1	0.62	0.37	0.61	0.39	0.59	0.35
21	0.78	0.55	0.58	0.37	0.57	0.34
34	0.77	0.56	0.56	0.40	0.59	0.36
56	0.80	0.56	0.55	0.33	0.57	0.31
100	0.76	0.64	0.55	0.41	0.57	0.34
Mean	*0.746*	*0.536*	*0.574*	*0.380*	*0.578*	*0.340*
SD	*0.072*	*0.100*	*0.029*	*0.032*	*0.011*	*0.019*

## Conclusion

Our analysis showed that the ARI scores reported in this article are inflated. The reported performance is based on ground-truth-informed model selection and does not constitute a fully label-independent evaluation of the method. The clustering accuracy does not surpass comparison methods when the epoch number is fixed. Selecting the model based on ground truth is impossible with datasets that do not include annotations and scientifically invalid. We suggest that the authors revise the reported clustering results, choose a more appropriate stopping strategy, and document this issue explicitly. This revision is very important and urgent to avoid misleading researchers who may adopt SpatialDG as a top-performing method.

**Key Points**

- SpatialDG’s reported accuracy is inflated due to ground-truth label leakage during evaluation.
- SpatialDG picked peak accuracy scores across training epochs by checking against known ground-truth labels.
- Evaluating fairly at the final epoch drops the median score significantly, failing to beat existing methods.
- Ground-truth selection also reduced performance variance across tissue samples and random seeds.

## Data Availability

The dorsolateral prefrontal cortex (DLPFC) dataset used in this paper is available at http://spatial.libd.org/spatialLIBD/.
